# Radiomics and machine learning for renal tumor subtype assessment using multiphase computed tomography in a multicenter setting

**DOI:** 10.1007/s00330-024-10731-6

**Published:** 2024-04-18

**Authors:** Annemarie Uhlig, Johannes Uhlig, Andreas Leha, Lorenz Biggemann, Sophie Bachanek, Michael Stöckle, Mathias Reichert, Joachim Lotz, Philip Zeuschner, Alexander Maßmann

**Affiliations:** 1 Department of Urology, University Medical Center Goettingen, Goettingen, Germany; 2 Department of Clinical and Interventional Radiology, University Medical Center Goettingen, Goettingen, Germany; 3 Department of Medical Statistics, University Medical Center Goettingen, Goettingen, Germany; 4 Department of Urology and Pediatric Urology, Saarland University, Homburg, Germany; 5 Department of Cardiac Imaging, University Medical Center Goettingen, Goettingen, Germany; 6 Department of Radiology and Nuclear Medicine, Robert-Bosch-Clinic, Stuttgart, Germany

**Keywords:** Renal cell carcinoma, Multidetector computed tomography, Radiomics, Computers, Machine learning

## Abstract

**Objectives:**

To distinguish histological subtypes of renal tumors using radiomic features and machine learning (ML) based on multiphase computed tomography (CT).

**Material and methods:**

Patients who underwent surgical treatment for renal tumors at two tertiary centers from 2012 to 2022 were included retrospectively. Preoperative arterial (corticomedullary) and venous (nephrogenic) phase CT scans from these centers, as well as from external imaging facilities, were manually segmented, and standardized radiomic features were extracted. Following preprocessing and addressing the class imbalance, a ML algorithm based on extreme gradient boosting trees (XGB) was employed to predict renal tumor subtypes using 10-fold cross-validation. The evaluation was conducted using the multiclass area under the receiver operating characteristic curve (AUC). Algorithms were trained on data from one center and independently tested on data from the other center.

**Results:**

The training cohort comprised *n* = 297 patients (64.3% clear cell renal cell cancer [RCC], 13.5% papillary renal cell carcinoma (pRCC), 7.4% chromophobe RCC, 9.4% oncocytomas, and 5.4% angiomyolipomas (AML)), and the testing cohort *n* = 121 patients (56.2%/16.5%/3.3%/21.5%/2.5%).

The XGB algorithm demonstrated a diagnostic performance of AUC = 0.81/0.64/0.8 for venous/arterial/combined contrast phase CT in the training cohort, and AUC = 0.75/0.67/0.75 in the independent testing cohort. In pairwise comparisons, the lowest diagnostic accuracy was evident for the identification of oncocytomas (AUC = 0.57–0.69), and the highest for the identification of AMLs (AUC = 0.9–0.94)

**Conclusion:**

Radiomic feature analyses can distinguish renal tumor subtypes on routinely acquired CTs, with oncocytomas being the hardest subtype to identify.

**Clinical relevance statement:**

Radiomic feature analyses yield robust results for renal tumor assessment on routine CTs. Although radiologists routinely rely on arterial phase CT for renal tumor assessment and operative planning, radiomic features derived from arterial phase did not improve the accuracy of renal tumor subtype identification in our cohort.

## Key Points

- *Radiomic feature analyses and ML algorithms can be utilized in routinely acquired CT studies from multiple imaging centers*.
- *Discrimination of renal tumor subtypes using CT-based radiomics and ML yields a moderate diagnostic performance (multiclass AUC* *=* *0.75 in independent testing data)*.
- *For renal tumor subtype discrimination using radiomic features, venous contrast phase CT yields the most pertinent imaging information, without evident diagnostic benefit of an added arterial contrast phase*.

## Introduction

In 2023, cancer of the kidneys and renal pelvis is estimated to account for 5% and 3% of incident cancer cases in men and women in the United States, respectively, most of which are renal cell carcinomas (RCC) [1]. A broader clinical utilization of radiological cross-sectional imaging and technical advancements has been discussed to contribute to the increasing incidence of RCC over the last decades, in particular for small renal tumors [2–4].

Renal tumors are pathologically classified according to their cellular origin and behavior, with each of these histological subtypes showing a specific profile regarding their incidence, clinical prognosis, and therapeutic options [5, 6]. Still, the radiological distinction of renal tumor subtypes on cross-sectional imaging is imperfect in clinical practice, with immediate ramifications for affected patients: for example, studies have shown that approximately 20% of renal tumors that were radiologically classified as malignant were in fact benign of histopathological assessment [7–9].

In clinical practice, dedicated CT protocols for renal tumor assessment are routinely performed using multiple contrast media (CM) phases including non-CM, arterial, and venous CM phases. While associated with a higher radiation dose than single-phase imaging, multiphase CT can identify varying CM enhancement patterns depending on renal tumor histology. For example, papillary RCC (pRCC) has been described to demonstrate low levels of peak enhancement and little enhancement fluctuation between CM phases, while rapid high enhancement in the arterial phase and CM washout is more suggestive for clear-cell RCC or oncocytoma [10–12]. Arterial phase imaging also provides additional information for planning, surgical procedures, or percutaneous renal tumor thermoablation, which has been shown to yield comparable outcomes to partial nephrectomy in small renal tumors [13]. For percutaneous renal tumor thermoablation in specific, interarterial CM application might aid in the guidance of ablation procedures, while additional interarterial embolization of renal tumors might improve outcomes [14, 15]. Still, the exact diagnostic benefit of multiphase CT over single-phase CT in the specific clinical setting of renal tumor subtype identification has not been fully evaluated so far.

An effective method for enhancing the assessment of renal tumors involves employing radiomic feature analyses and machine learning (ML) algorithms, which have shown strong performance across different classification tasks and types of medical imaging [16, 17]. One earlier study from our research group utilized these techniques to evaluate renal tumor subtype on venous CM phase CTs with an AUC = 0.72 [18]. Still, the aforementioned study and most others published on this topic did not include a comparison of multiple CM-phases or CT scans from different imaging centers or lacked external validation [19–21].

Thus, the study presented here seeks to evaluate renal tumor subtypes through the application of radiomic feature analyses and subsequent ML. This analysis utilizes a multicenter dataset comprising multiphase CT studies collected from routine clinical practice.

## Material and methods

This retrospective study received prior approval by the local ethics committees (No 2/4/17 and No 67/19) and is compliant with the Declaration of Helsinki.

### Study cohort selection

The inclusion criteria comprised adult patients with renal tumors who underwent surgical resection between 2012 and 2022 at either (1) the Department of Urology, University Medical Center Goettingen, or (2) the Department of Urology and Pediatric Urology, Saarland University. All patients had undergone preoperative contrast-enhanced CT imaging in both arterial (corticomedullary) and venous (nephrogenic) phases at these tertiary centers or external imaging facilities (such as radiological private practices or other hospitals). The analysis was limited to patients diagnosed with clear cell, papillary, or chromophobe RCC, oncocytoma, or angiomyolipoma (AML), regardless of tumor size.

Patients exhibiting diffusely infiltrative tumors, such as lymphoma or those with chronic inflammatory changes as well as those presenting with cystic neoplasms were excluded. Renal tumor patients, for whom the reference standard of histological assessment was not available (i.e., those with fat-rich AMLs on CT) were excluded as well.

### CT imaging

Arterial and venous CM-phase CT studies obtained in the two tertiary centers and outside imaging centers were included, without restrictions regarding acquisition protocol, CT scanner, slice thickness, or imaging artifacts, to reflect clinical heterogeneity in CT imaging acquisition and quality.

The renal CT imaging protocol at the two tertiary centers comprised: (i) a non-contrast scan; (ii) an arterial-phase scan with bodyweight adapted intravenous administration of iodinated contrast media (1.1 mL Ultravist 370/kg) followed by a 30 mL NaCl chaser (both at 3 mL/s), delay of 10 s after enhancement > 120 Hounsfield Units in the abdominal aorta; (iii) venous-phase scan 40 s after arterial-phase scan.

### Radiomic feature analyses

Renal tumor segmentation and radiomic feature analyses were conducted using the open-source software 3D Slicer [22]. The radiomics features derived in this study adhered to an internationally standardized and reproducible approach, following feature definitions outlined by the Imaging Biomarker Standardization Initiative [23, 24]. These features encompass renal tumor voxel intensities, and 2D and 3D shape characteristics, along with higher-order features, as detailed in the supplementary material. A bin width of 25 was selected for the radiomic feature analyses.

Renal tumors were manually segmented by an experienced GU-radiologist with delineation of the region of interest (ROI) on all axial arterial and venous phase CT slices. The number of CT slices varied according to the size of renal tumors and the CT slice thickness.

### Renal tumor assessment

The histological reference standard for all renal tumors was established by histopathological analyses at the Department of Pathology at both participating tertiary centers evaluating partial or radical nephrectomy specimens.

### Machine learning

For statistical modeling, data from one tertiary center was used as the training dataset, and data from the other tertiary center as an independent (outside) testing dataset. All analyses were performed separately using radiomics from arterial, venous, as well as combined arterial + venous CM-phase CTs.

Radiomic feature preprocessing comprised: (i) omitting variables with so-called “near zero variance”, whereby only one representative variable was retained; and (ii) omitting variables with linear dependencies. After preprocessing, a total of 127 radiomic features + age and gender were considered for further evaluation.

A 10-fold cross-validation (CV) was used for internal validation and assessment of the generalizability. Stratified sampling was implemented to maintain the distribution of renal tumor subtypes in all training and testing subsets.

The steps detailed below were applied within each CV-fold and CV-repetition to avoid the leakage of information.

Class imbalance was handled using: (i) no class imbalance handling, (ii) SMOTE with nearest-neighbor-based oversampling of minority classes [25], and (iii) weighting.

Feature selection was applied using: (i) no feature selection technique (passing all features for downstream ML); (ii) recursive feature elimination (RFE), where a random forest model ordered features according to their importance in a classifier, retaining only the top features with the best performance. To mitigate bias, RFE was performed using an internal 10-fold CV [26]; and (iii) principal component analysis (PCA) where within each fold, PCA was calculated on the training set, retaining components that explained 80% of the total variance, and projecting the rest samples onto the same PCA.

Using the methodology detailed above, an extreme gradient boosting tree classification model (XGB) was trained.

### Statistical analyses and diagnostic performance assessment

The XGB algorithms diagnostic accuracy was evaluated using the area under the curve (AUC) with a generalization to multiclass problems that implements all pairwise comparisons [27]. Diagnostic performance measures reported in this study were calculated from the out-of-bag samples of the 10-fold cross-validation for all CM-phases. Diagnostic performance was validated on an independent testing dataset from one tertiary center. The Youden-Index was used to define cut-off values for calculation of sensitivity and specificity.

Statistical analyses were conducted utilizing R and RStudio, employing the R package “caret” [28]. A significance level of 0.05 was selected, and all reported *p* values are two-sided.

## Results

### Study cohort

**Fig. 1:**
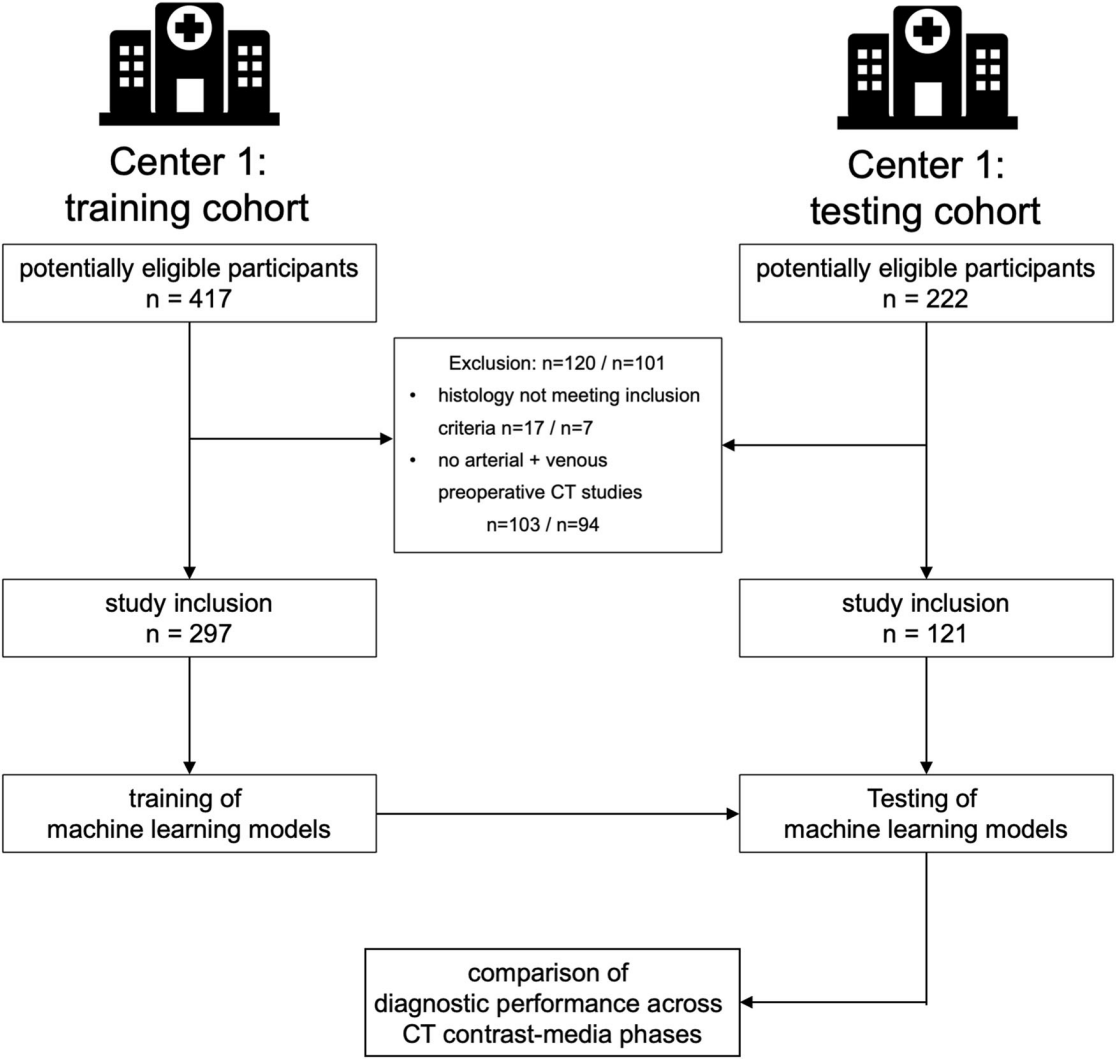
Study flowchart showing patient inclusion and exclusion

The training dataset comprised *n* = 297 patients and the validation dataset *n* = 121 patients. A study flowchart is provided in Fig. 1.

As detailed in Table 1, the training dataset showed a larger proportion of female patients compared to the validation dataset, although statistical significance was not reached (38.4% vs 29.8%, *p* = 0.12). Similarly, no statistically significant difference was evident in the age distribution across centers (mean 65 ± 12 vs. 64 ± 13 years, *p* = 0.40). The majority of CT studies included in this study were acquired at outside imaging centers, although the proportion was higher in the training compared to the testing dataset (18.7% vs. 3.2%, *p* < 0.001).

**Table 1:** Characteristics of included patients and renal tumors

Parameter	Level	Training dataset	Test dataset	*p* value
*n*		297	121	
sex				0.12
	f	114 (38.4%)	36 (29.8%)	
	m	183 (61.6%)	85 (70.2%)	
Age [y]				0.4
	mean ± sd	65 ± 12	64 ± 13	
Subtype				0.01
	AML	16 (5.4%)	3 (2.5%)	
	ccRCC	191 (64.3%)	68 (56.2%)	
	chRCC	22 (7.4%)	4 (3.3%)	
	oncocytoma	28 (9.4%)	26 (21.5%)	
	pRCC	40 (13.5%)	20 (16.5%)	
Tumor diameter [mm]				< 0.01
	mean ± sd	62 ± 36	42 ± 16	

*AML* angiomyolipoma, *ccRCC* clear cell renal cell carcinoma, *chRCC* chromophobe renal cell carcinoma, *pRCC* papillary renal cell carcinoma

The distribution of renal tumor subtypes demonstrated statistically significant variability (overall difference *p* = 0.01): while clear cell RCC (ccRCC) was the predominant renal tumor in both datasets (64.3% and 56.2%), AML was more frequent in the training dataset (5.4% vs 2.5%), and oncocytoma more frequent in the testing dataset (9.4% vs 21.5%).

**Fig. 2:**
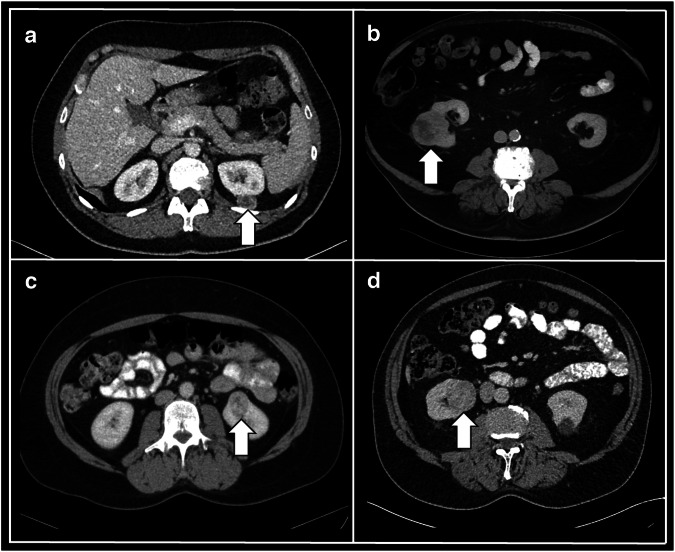
Representative CT case studies. **a** 49-year-old female patient with left-sided renal tumor (arrow), histologically identified as AML; **b** 70-year-old male patient with right-sided renal tumor (arrow), histologically identified as ccRCC; **c** 49-year-old female patient with left-sided renal tumor (arrow), histologically identified as oncocytoma; **d** 68-year-old male patient with right-sided renal tumor (arrow), histologically identified as pRCC

The mean 3-dimensional (3D) diameter of included renal tumors was 62 ± 36 mm in the training and 42 ± 16 mm in the testing dataset (*p* < 0.01). Representative patient cases are provided in Fig. 2.

### Machine learning algorithms: training

Across the out-of-bag CV samples in the training dataset, the diagnostic accuracy of the XGB algorithm was highest using SMOTE and no feature selection for combined CM-phases and was thus selected as the reference for further downstream modeling. For prediction of the renal mass histological subtypes, the XGB reached a multiclass AUC = 0.8 for combined CM-phases; 0.64 for arterial CM-phase; and 0.81 for venous CM-phase.

**Fig. 3:**
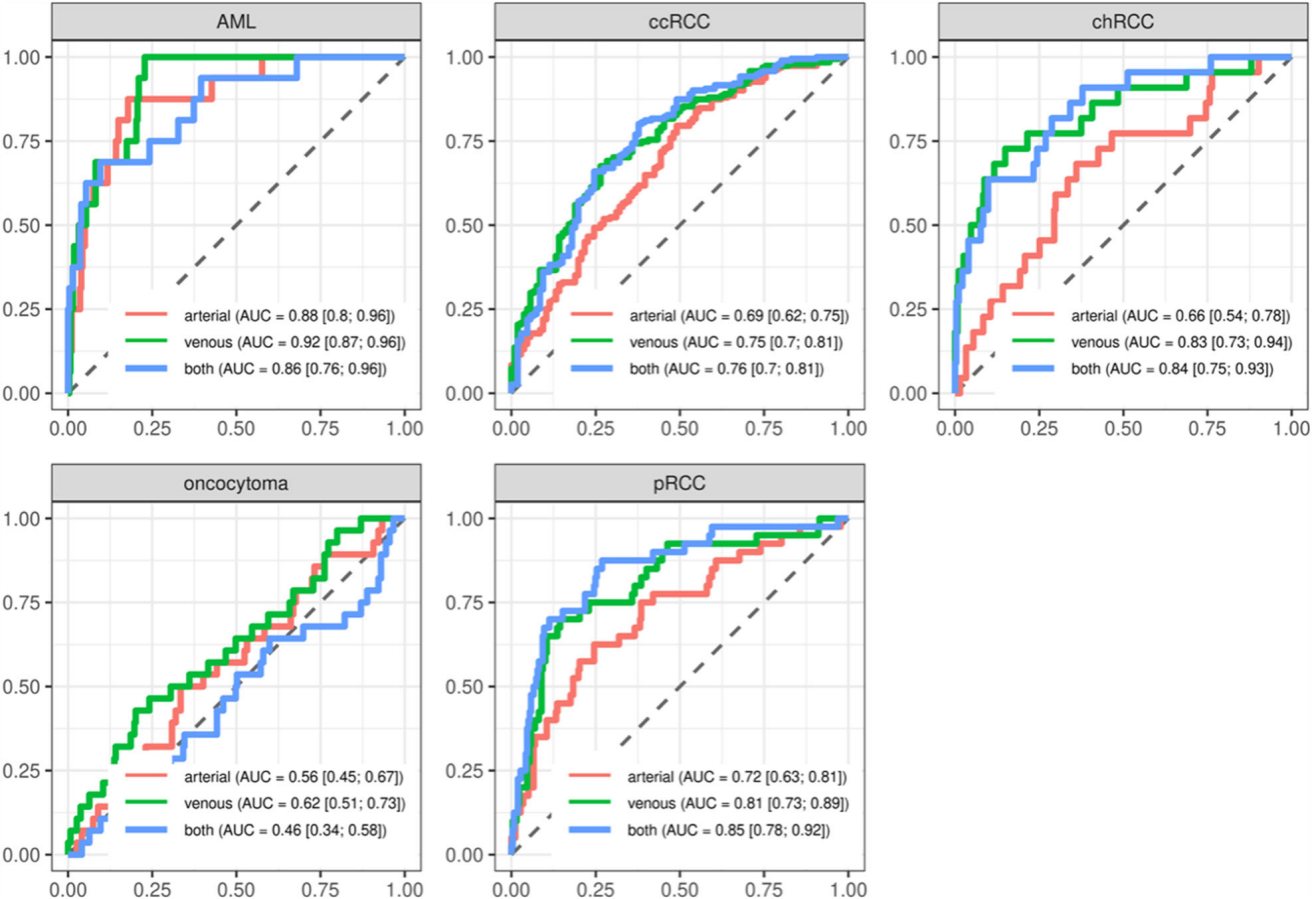
ROC curves for differentiation of specific renal tumor subtypes from all other subtypes across different contrast media phases (results from out-of-bag cross-validation samples). AML angiomyolipoma, ccRCC clear cell renal cell carcinoma, chRCC chromophobe renal cell carcinoma, pRCC papillary renal cell carcinoma

Table 2 summarizes the corresponding diagnostic accuracy (measured as AUC and sensitivity/specificity) for pairwise comparisons of one specific histological subtype versus the remaining histological subtypes. The highest AUC was consistently achieved for differentiation of AML (AUC = 0.90–0.94; versus any other renal tumor). The lowest AUC was evident for differentiation of oncocytoma (AUC = 0.57–0.69). Associated ROC curves are depicted in Fig. 3.

**Table 2:** Diagnostic performance for specific renal tumor subtype detection (against all other subtypes) according to CT contrast-media phase

	Combined CM-phases		Arterial CM-phase		Venous CM-phase	
	Sensitivity/specificity	AUC	Sensitivity/specificity	AUC	Sensitivity/specificity	AUC
AML	0.40/0.99	0.94	0.20/0.99	0.90	0.35/0.99	0.93
ccRCC	0.87/0.49	0.75	0.86/0.41	0.70	0.84/0.47	0.78
chRCC	0.35/0.97	0.87	0.13/0.95	0.71	0.38/0.97	0.9
oncocytoma	0.08/0.95	0.65	0.00/0.95	0.57	0.15/0.95	0.69
pRCC	0.48/0.94	0.88	0.35/0.93	0.77	0.40/0.92	0.80

*AML* angiomyolipoma, *ccRCC* clear cell renal cell carcinoma, *chRCC* chromophobe renal cell carcinoma, *pRCC* papillary renal cell carcinoma

### Machine learning algorithms: testing

**Fig. 4:**
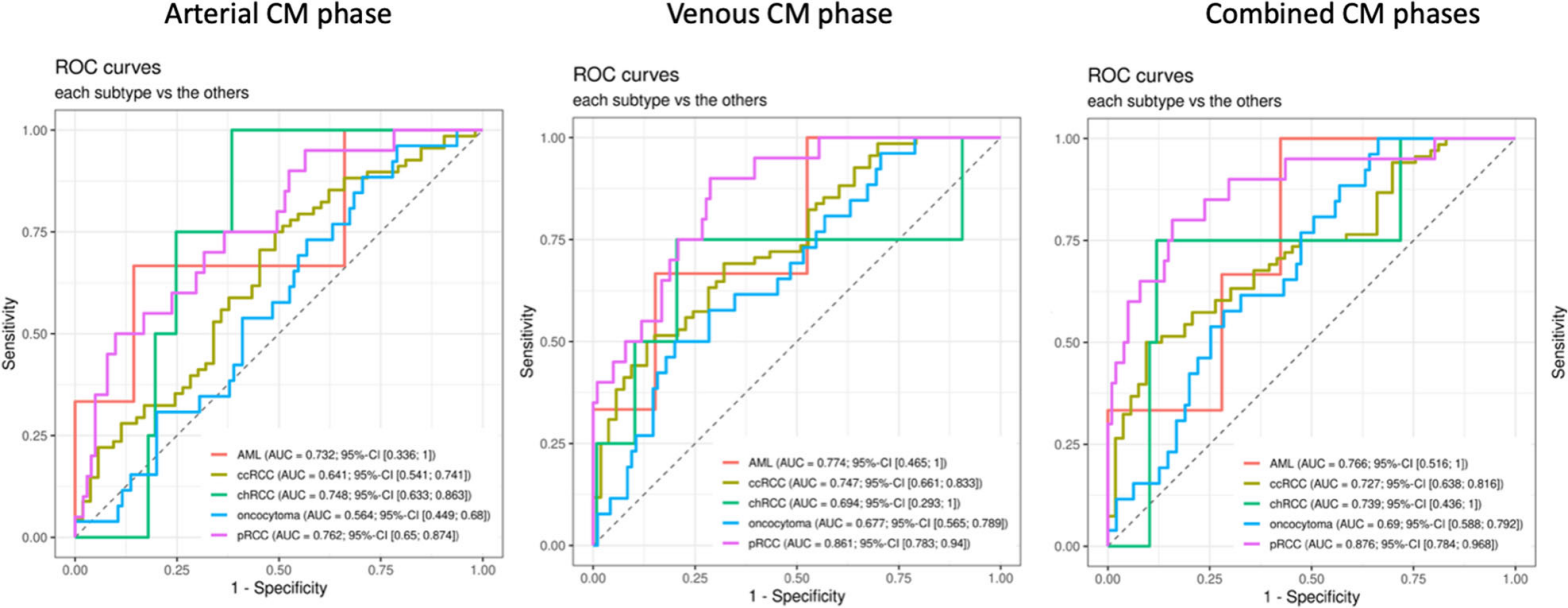
ROC-curves for identification of specific renal tumor subtypes (against all other subtypes) in the testing dataset. AML angiomyolipoma, ccRCC clear cell renal cell carcinoma, chRCC chromophobe renal cell carcinoma, pRCC papillary renal cell carcinoma

The application of the XGB algorithm (with SMOTE and without feature selection) in the independent testing dataset yielded an AUC = 0.75, AUC = 0.67, and AUC = 0.75, for combined, arterial, and venous CM-phase CTs, respectively. The corresponding ROC curves are depicted in Fig. 4. The performance to identify specific subtypes among different contrast media phases is summarized in Table 3.

**Table 3:** Diagnostic performance for identification of specific renal tumor subtypes in the testing dataset, comparing different contrast media (CM) phases

Renal tumor subtype	roc1	auc1	roc2	auc2	*p* value
AML	combined CM phases	0.7655	arterial	0.7316	0.761
AML	venous	0.774	arterial	0.7316	0.418
AML	venous	0.774	combined CM phases	0.7655	0.90
ccRCC	combined CM phases	0.727	arterial	0.641	0.068
ccRCC	venous	0.7467	arterial	0.641	0.06
ccRCC	venous	0.7467	combined CM phases	0.727	0.544
chRCC	combined CM phases	0.7393	arterial	0.7479	0.94
chRCC	venous	0.6944	arterial	0.7479	0.742
chRCC	venous	0.6944	combined CM phases	0.7393	0.47
oncocytoma	combined CM phases	0.6903	arterial	0.5644	0.024
oncocytoma	venous	0.6773	arterial	0.5644	0.098
oncocytoma	venous	0.6773	combined CM phases	0.6903	0.709
pRCC	combined CM phases	0.8762	arterial	0.7619	0.022
pRCC	venous	0.8614	arterial	0.7619	0.045
pRCC	venous	0.8614	combined CM phases	0.8762	0.654

*AML* angiomyolipoma, *ccRCC* clear cell renal cell carcinoma, *chRCC* chromophobe renal cell carcinoma, *pRCC* papillary renal cell carcinoma

## Discussion

Multiphase CT is the radiological mainstay of renal tumor assessment and aids in the planning of surgical and thermoablation procedures. While some renal tumors, such as fat-rich AMLs with macroscopic fat, can be confidently classified on CT, the identification of the other renal tumor subtypes remains a radiological challenge. Consequently, up to 20% of renal tumors that were radiologically classified as malignant turn out to be benign on histological assessment after surgical resection, resulting in a surgical overtreatment [7–9]. To date, the diagnostic benefit of multiphase CT has so far not been systematically evaluated in the context of AI-aided renal tumor assessment and using CT studies from multiple centers.

This study evaluated a large-scale cohort of renal tumor patients with multiphase CT from multiple imaging centers and provided an independent external dataset for statistical testing. Different CT scanners with varying slice thickness as well as CT studies with imaging artifacts were included to better reflect the diversity of CT studies that are clinically encountered by radiologists. All renal tumor specimens evaluated in this study underwent histopathological assessment to establish a precise reference standard. In this clinical dataset, radiomic feature analyses and ML algorithms were utilized to predict renal tumor subtypes using preoperative CT imaging.

The demographic characteristics of included patients are in line with the recent literature, showing a male predominance and peak incidence of renal tumors between age 60 and 70 [4, 5]. Interestingly, the frequency of specific renal tumor subtypes varied according to the accruing center, highlighting the heterogeneity encountered when assessing different patient cohorts and corroborating the need for external model testing. In general, the frequency of benign renal tumors in our study (14.8–24%) was comparable to the literature, reporting ranges between 20% to 30% [8, 29].

Using an XGB algorithm, we achieved an AUC = 0.84 in the internal validation dataset for the discrimination of different renal tumor subtypes using combined arterial + venous CM-phases. On the independent testing dataset, the algorithm yielded an AUC = 0.75. Using radiomic features from the venous CM-phase only, the XGB demonstrated an AUC = 0.75 in the testing dataset, and arterial CM-phase an AUC = 0.67. These results indicate that radiomic features derived from venous CM-phase reflect the most crucial imaging characteristics of different renal tumor subtypes. It also challenges the diagnostic benefit of an added arterial CM-phase for renal tumor subtyping.

Noticeably, the XGB algorithm performed better in our training cohort than in the independent testing dataset (AUC = 0.84 vs. AUC = 0.75). These discrepancies might be attributed to statistical overfitting that was not fully addressed using multifold cross-validation. Still, center-specific differences in the distribution of renal tumor subtypes and renal tumor diameter, as well as varying acquisition protocols and the high proportion of CT studies acquired from outside imaging centers in the testing cohort could have contributed as well. Overall, the center-specific diagnostic performance of our radiomics-based XGB algorithm shows the challenges that need to be addressed when implementing imaging-based ML models on data from clinical practice and the potential necessity for algorithmic adaptation at each clinical site. Further, as suggested by other authors, the combination of AI approaches with expert radiologist knowledge might further stabilize and improve their diagnostic performance [30].

In both our training and testing datasets, the identification of oncocytomas was most challenging for the XGB algorithm, demonstrating the lowest AUCs irrespective of CM phases. These results might reflect the similarities in radiological appearance between oncocytomas and ccRCC, presenting with a morphologically similar central scar and central necrosis, respectively.

Notably, the diagnostic performance for assessing renal tumor subtypes in this study is inferior to the discrimination reported for benign versus malignant renal tumors (AUC = 0.83) in a previous publication by our research team [31]. Other studies on renal tumor subtype assessment revealed diverging results. Evaluating different renal tumor subtypes, Coy et al used peak lesion attenuation analyses, which yielded pairwise AUCs ranging between 0.96 and 0.79 [19]. Similar to the results of our study, the diagnostic performance reported by Coy et al was worse for the identification of oncocytomas (AUC = 0.79) and fat-poor AMLs (AUC = 0.83). Using a standard logistic regression without cross-validation or external testing, Sasguri and colleagues evaluated to diagnostic performance of CT attenuation values and skewness from biphasic contrast CT to discriminate oncocytomas from other renal tumors, reporting an AUC = 0.8 [32]. A recent meta-analysis by Firouzabadi et al corroborates a high heterogeneity of radiomic feature analyses for the assessment of renal oncocytomas among included studies, reporting a pooled sensitivity and specificity of 0.82 and 0.8 [33]. Given the heterogeneity and diagnostic uncertainty in renal tumor subtype assessment, in particular, regarding oncocytomas, it would be beneficial to establish a diagnostic baseline by visual assessment and renal tumor subtyping by radiologists. Unfortunately, as CT studies and associated histology were known to the involved radiologists, a post-hoc blinded renal tumor assessment was not possible within the scope of the presented study.

The higher diagnostic performance of the aforementioned studies might have resulted from the utilization of standardized renal tumor CT acquisition protocols at one imaging center. On the contrary, the CT studies included in our study were obtained in a multicenter setting, including different CT scanners, variable slice thickness, and studies with imaging artifacts. Especially given the independent testing performed, our results might be more generalizable and realistic in a clinical scenario.

Recent meta-analyses and review articles have summarized the literature on ML for renal tumor assessment [34, 35]. For example, Feng et al evaluated *n* = 58 patients to distinguish RCCs and fat-poor AMLs, achieving an accuracy of approximately 94% [20]. Kocak et al reported an accuracy of 85% in discriminating ccRCC and non-clear-cell RCC in 68 patients with a lower accuracy for renal tumor subtype assessment (69%) [21]. An AUC of up to 92% for selected renal tumor subtypes was achieved by Yu et al in 119 patients using radiomic features and SVMs, although the authors failed to comprehensively assess all tumor subtypes in one global model [17].

The aforementioned studies yield promising results but might be limited in their generalizability, given their focus on single-center CT studies of high quality and lack of external, independent testing. Still, suboptimal renal tumor CT studies are routinely encountered in radiological clinical practice given that referral patterns might result in urological patients that present with CT studies from external imaging centers.

Our study is not devoid of limitations. First, patients were accrued at two tertiary urological referral centers in Germany, which might limit the generalizability of our findings to a non-Caucasion population. Second, our analyses were restricted to the five most common renal tumor subtypes, thus not reflecting the diversity of renal neoplasms (i.e., cystic renal masses) encountered in clinical routine. This limits the a-priori applicability of the presented methods for individual patients. Third, due to the inclusion of patients with histopathologically assessed renal tumors in this study, patients with fat-rich AMLs that have been correctly identified on CT studies by radiologists were excluded. In a clinical cohort without this patient preselection, the presented methods might therefore yield different results. Finally, renal tumor subtypes have not been evaluated by radiologists in this study, which would have provided a comparative measure for the diagnostic performance of the ML algorithm.

## Conclusions

Radiomic feature analyses acquired from clinical routine CT yielded robust results for renal tumor assessment on a large-scale independent testing dataset. Although radiologists routinely rely on arterial phase CT for renal tumor assessment and operative planning, radiomic features derived from arterial phase did not improve the accuracy of renal tumor subtype identification in our cohort using an XGB algorithm.

Given the results of this study, among all renal tumors, oncocytomas are the hardest to differentiate using CT.

## Supplementary information

Supplementary Material

## List of abbreviations

AML: Angiomyolipoma
AUC: Area under the curve
ccRCC: Clear cell RCC
chRCC: Chromophobe RCC
CM: Contrast media
CV: Cross-validation
ML: Machine learning
PCA: Principal component anylsis
pRCC: Papillary RCC
RCC: Renal cell carcinoma
RFE: Recursive feature elimination
ROC-curve: Receiver operating characteristics curve
XGB: Extreme gradient boosting tree
