# Supplementary material for: Radiomics and machine learning for renal tumor subtype assessment using multiphase computed tomography in a multicenter setting
Source: Eur Radiol. 2024 Apr 18;34(10):6254–63. doi: 10.1007/s00330-024-10731-6 (PMC11399155; doi:10.1007/s00330-024-10731-6)
Supplement: Supplementary file 1 — Supplementary Material [file 330_2024_10731_MOESM1_ESM.pdf]

Radiomics and machine learning for renal tumor subtype assessment using multiphase computed tomography in a multicenter setting

ELECTRONIC SUPPLEMENTARY MATERIAL

Details on evaluated radiomic feature analyses

Radiomic feature analyses were conducted according to 8 subclasses:

1. first-order statistic (describing renal mass voxel intensity)
2. 3D shape features (describing 3-dimensional size and shape of renal mass)
3. 2D shape features (describing 2-dimensional size and shape of renal mass)
4. gray level co-occurrence matrix features (GLCM; describing second-order joint probability function of renal mass)
5. gray level size zone matrix features (GLSZM; quantifying gray level zones in renal mass)
6. gray level run length matrix features (GLRLM; quantifying gray level runs in renal mass)
7. neighboring gray tone difference matrix features (NGTDM; quantifying the difference between a gray value and average gray value of its neighbors in renal mass)
8. gray level dependence matrix features (GLDM; quantifying gray level dependencies of renal mass).

In addition, information on renal tumor size, number of voxels, spacing, and center of mass was extracted based on original images, mask-originals, and mask-corrected images. All radiomic features were one-dimensional for downstream use in machine learning models. The full feature vector consisted of 127 radiomics features plus age and gender.

Details on evaluated machine learning algorithm

Extreme gradient boosting (XG boost): tree-based optimization and boosting model. The number of boosting iterations (*nrounds*) was set to 100 with early stopping allowed after 3 iterations without performance improvement. The maximum tree depth (*max_depth*), the shrinkage (eta*)* to control learning rate, and the minimum sum of instance weight (*min_child_weight*) to indicate the minimum number of observations in a terminal node were optimized using an internal CV.
